# The diagnosis of hypophosphatasia in children as a multidisciplinary effort: an expert opinion

**DOI:** 10.1007/s40618-023-02199-w

**Published:** 2023-09-26

**Authors:** G. I. Baroncelli, G. Carlucci, E. Freri, M. R. Giuca, V. Guarnieri, G. Navarra, B. Toschi, S. Mora

**Affiliations:** 1 Pediatric and Adolescent Endocrinology, Division of Pediatrics, Department of Obstetrics, Gynecology and Pediatrics, University Hospital, Pisa, Italy; 2 OPT S.P.A., Soluzioni Per Il Mondo Healthcare, Milan, Italy; 3 Department of Pediatric Neuroscience, Fondazione IRCCS Istituto Neurologico Carlo Besta, Milan, Italy; 4 Unit of Pediatric Dentistry, Department of Surgical Medical Molecular Pathology and Critical Area, Dental and Oral Surgery Clinic, University of Pisa, Pisa, Italy; 5 Division of Medical Genetics, Fondazione IRCCS Casa Sollievo della Sofferenza, Foggia, Italy; 6 Section of Medical Genetics, Department of Medical and Oncological Area, University Hospital, Pisa, Italy; 7 Laboratory of Pediatric Endocrinology, Department of Pediatrics, IRCCS San Raffaele Hospital, Milan, Italy

## Abstract

Hypophosphatasia (HPP) is a rare genetic disorder in which pathogenic variants of the *ALPL* gene lead to a marked decrease of tissue non-specific alkaline phosphatase (TNSALP) activity. Although HPP is a systemic disorder, its clinical manifestations are more evident on bones, teeth, muscle and central nervous system. The clinical spectrum ranges from severe forms with extreme skeletal deformities, respiratory impairment, seizures, to very mild forms with onset in late adulthood and few clinical signs. The diagnosis can be suspected by measurement of TNSALP activity, but the insufficient awareness among health professionals and the lack of official guidelines are responsible for delayed diagnosis in children with HPP. The purpose of the current document is to provide an expert opinion directed at optimizing the diagnostic pathway of pediatric HPP. From April to December 2022, a multidisciplinary working group of 6 experts including two pediatric endocrinologists, a pediatric neurologist, a pediatric odontologist, a clinical geneticist, and a molecular biologist gathered in a series of periodic meetings to discuss the main issues related to the diagnosis of HPP in children and formalize an Expert Opinion statement. The experts agreed on a diagnostic trail that begins with the recognition of specific clinical signs, leading to biochemical analyses of TNSALP activity and vitamin B6 serum concentration. Very important are the neurological and dental manifestation of the disease that should be thoroughly investigated. The evaluation of TNSALP activity must consider sex and age variability and low activity must be persistent. Repeated blood measurements are thus necessary. The molecular analysis is then mandatory to confirm the diagnosis and for genetic counseling.

## Background

Hypophosphatasia (HPP—OMIM146300, 241,500, 241,510) is a genetic disorder with very heterogeneous clinical manifestations. HPP is due to loss-of-function variants in the *ALPL* gene encoding tissue non-specific alkaline phosphatase (TNSALP) [1]. To date, more than 400 different pathogenic variants have been described, showing a high degree of allelic heterogeneity of the disease [2].

TNSALP is present in all tissues, but it is abundantly expressed in liver, kidney and bone [3]. Its function is to release phosphate from several substrates in an alkaline medium. In *vivo*, TNSALP catalyzes the hydrolysis of pyrophosphate (PPi), releasing phosphate, which becomes available for the formation of hydroxyapatite crystals. The accumulation of PPi due to inactive TNSALP leads to defective bone mineralization, as PPi directly binds to growing crystals and because it is a potent stimulator of the synthesis of osteopontin, which in turn has an inhibitory effect on osteoblasts [4, 5]. Pyridoxal-5'-phosphate (PLP, a circulating form of vitamin B6) is a second substrate of TNSALP. PLP is essential for the formation of neurotransmitters [6], but it cannot enter the cell in its form. The release of phosphate by the action of TNSALP converts PLP into pyridoxal (PL), which is able to cross the cell membrane. PL is then transformed back into PLP in neurons [7, 8]. The impairment of TNSALP activity leads to accumulation of circulating substrate and a deficiency of intracellular PLP in neurons. The third substrate found in vivo is phosphoetanolamine (PEA). Although its role is yet unclear, TNSALP deficiency is associated to elevated serum and urine concentration of PEA [9].

The clinical spectrum of HPP is extremely variable, ranging from severe, sometimes lethal, perinatal forms, to dental complication only in children and adults, to a very mild form with onset in late adulthood. A classification based on the age at diagnosis is widely used [1], but recently a new nosology based on inheritance has been proposed [10]. The prevalence of the severe forms is estimated to be 1/300,000 [11], while mild forms have a prevalence of 1/508 [10]. Table 1 summarizes the clinical manifestations of the HPP forms. All forms share various degree of bone mineralization deficit, and they are characterized by reduced serum alkaline phosphatase (ALP) activity, a hallmark of *ALPL* variants [12]. The heterogeneity of the clinical signs and the lack of awareness among healthcare professionals complicates the diagnosis of the disease, which is often delayed both in children and adults. A recent study reported a median delay of 12 months from the earliest HPP manifestation in children, but in some instances, the delay reached several years [13].

**Table 1:** Subtypes and clinical features of HPP

HPP forms	Prevalence	Transmission	Age at onset	Clinical features
Severe	1:300,000	Autosomal recessive	Perinatal, Infant	Severe bone mineralization deficiency with bowing of limbs, small chest, pulmonary hypoplasia, and muscle weakness resulting in the need for ventilator support Neurological involvement including irritability and pyridoxine-dependent seizures
Moderate	1:2430	Autosomal recessive or autosomal dominant	Child, Adult	Rickets, fractures, short stature and poor mobility Craniosynostosis and increased intracranial pressure Premature loss of deciduous teeth Premature loss of primary and secondary teeth with intact roots in adults Musculoskeletal pains Fragility fractures Chondrocalcinosis and pseudogout
Mild	1:508	Autosomal dominant	Adult	Arthromyalgia Microcrystal arthropathy Fragility fractures in adulthood

The prognosis of the severe form is poor, with a low survival rate [14], whereas the prognosis of the milder forms is more benign [15]. Nevertheless, rickets and osteomalacia, bone deformities, muscular weakness, dental and neurological problems may remarkably impact the quality of life of the patients. A timely diagnosis is, therefore, important to reduce the burden of the disease.

Conventional management in HPP is driven by the major symptoms and signs of each affected individual. Since 2015, a targeted treatment is available, based on the administration of asfotase alpha (Strensiq®, Alexion Pharmaceuticals Inc, Boston, MA, USA), a bone-targeted, human recombinant TNSALP [16, 17]. Asfotase alpha is a soluble protein consisting of the catalytic domain of human TNSALP, the human immunoglobulin G1 Fc region (which function is to prolong the circulating half-life), and a deca-aspartate peptide domain for hydroxyapatite targeting [16, 17]. The treatment has been shown to be extremely well tolerated with few side effects, and it is very effective [17–23].

A timely diagnosis of HPP is extremely important to prevent the clinical problems associated. Apparently simple, the diagnostic pathway is not straightforward in real life. HPP may be confused with other more frequent diseases, and the biochemical features may be overlooked. To date, there are not official guidelines for the diagnosis of HPP. We gathered a multidisciplinary group of experts to identify the main signs and symptoms that can provide the basis for an efficient approach for the early diagnosis of HPP in children.

## Methods

From April to December 2022, a multidisciplinary working group of six Italian experts including two pediatric endocrinologists, a pediatric neurologist, a pediatric odontologist, a clinical geneticist, and a molecular biologist gathered in a series of periodic meetings to discuss the main issues related to the diagnosis of HPP in children and formalize an Expert Opinion statement, starting from the analysis of the main available literature evidence on four topics: biochemistry, neurological involvement, dental issues, and the role of genetics.

A Scientific Literature Review was performed in Pubmed to identify the relevant studies. Manual searches of key word strings were performed for each one of the main topic. To identify publications that met the inclusion criteria, the members of the multidisciplinary group critically reviewed all the full text articles included in the first-pass screening to verify if they carried relevant data and information to the respective study topics. A final list of references was compiled for inclusion.

Following the Scientific Literature Review, the multidisciplinary working group drafted, refined and agreed on an Expert Opinion statement.

This study does not involve the participation of human subjects and, therefore, no ethical approval has been requested.

## Diagnostic hallmarks

### Biochemistry

Despite the absence of current approved guidelines by the scientific community for the diagnosis of HPP in children, the scientific literature indicates that clinical characteristics, blood measurements of some biochemical parameters, and genetic testing for mutations in the *ALPL* gene can confirm the diagnostic suspicion of HPP.

In particular, biochemical parameters play a fundamental role in the diagnostic process. The measurement of ALP activity represents the hallmark for the diagnosis of HPP. Blood and urine tests for the concentration of the substrates of ALP represent a completion of the diagnostic path. The measurement of PPi, although very informative, is currently performed only in research setting. We, therefore, concentrated on the other substrates, PLP and PEA.

#### Alkaline phosphatase (ALP)

Although the measurement of ALP activity is one of the most widely employed test in clinical laboratory and the analytical procedures are mostly automated, there are issues that should be considered to prevent interpretation errors.

Pre-analytical problems may arise if blood samples are not collected properly, as the enzyme may be deactivated by the presence of Mg^2+^ or Zn^2+^, citrate or EDTA [5].

Each assay differs in analytic conditions and thus produces results that are not directly comparable to those obtained with a different method [24]. For this reason, it is important to rely on assay-specific reference values.

The activity of ALP changes as a function of age, sex, and pubertal status in growing individuals [25–30]. The bone isoform of ALP is prevalent in childhood and adolescence, and its activity reflects the changes occurring during growth [31]. As a result, normal limits vary during the developmental age and are higher in children than in adults [32]. In case of HPP, ALP activity is persistently subnormal compared to the ranges proposed for age and sex, and a common mistake is to compare the values obtained in a child to reference for adult individuals.

Low measurements of ALP are not uncommon. Therefore, the assessment of patients with reduced ALP activity requires the exclusion of other conditions that may be associated with this alteration. Table 2 summarizes the main conditions that should be considered in the differential diagnosis of HPP.

**Table 2:** Main causes of hypophosphatasaemia

Clofibrate therapy	Folic acid deficiency
Bisphosphonate therapy	Zn^2+^ deficiency
Denosumab therapy	Mg^2+^ deficiency
Corticosteroid therapy	Cleidocranial dysplasia
Estrogen therapy	Type II osteogenesis imperfecta
Vitamin D toxicity	Wilson's disease
Radioactive heavy metals toxicity	Cushing's syndrome
Cardiac surgery with bypass	Hypothyroidism
Celiac disease	Hypoparathyroidism
Pernicious or severe anemia	Massive transfusions
Malnutrition	Milk-alkali syndrome
Vitamin C deficiency	Multiple myeloma
Vitamin B12 deficiency	

Repeated blood samplings are necessary to confirm persistently subnormal ALP activity, as a single blood test is not sufficient to diagnose or exclude HPP.

#### Pyridoxal-5’-phosphate (PLP)

Recent studies show that PLP appears to have higher sensitivity and specificity compared to other substrates, such as PEA, for the diagnosis of HPP and also its concentration correlates with disease severity [33].

PLP is quantitatively the most important form of vitamin B6 in plasma and is a direct indicator of vitamin B6 activity. After blood collection from the patient, if correctly stored avoiding direct exposure of the sample to light, PLP is stable for 24 h at 4–8 °C or at room temperature.

There are few data related to the definition of reference values both for the adult population and for the pediatric population. A study conducted on 120 healthy blood donors aged > 20 years proposed normal range values (2.5–97.5 percentile) for sex classes: 20.8–176 nmol/L for females and 24.7–278 nmol/L for males [34]. The HELENA study (HealthyLifestyle in Europe by Nutrition in Adolescence) contributed to the definition of reference values in populations of adolescents, observing variations in PLP levels in a random sample of 1051 European adolescents aged between 12.5 and 17.5 [35]. The results showed that concentrations were on average higher in boys than in girls (66.3 vs 60.6 nmol/L), with an increasing trend as age increased in both sex classes. However, further studies are still needed to confirm the reference limits in younger populations. The measurement of circulating vitamin B6 concentration is mandatory in case of neurological involvement, considering that it is a potentially treatable condition, specifically through an acute supplementation of 100 mg IV during EEG recording [36].

To exclude the possibility of interference on the biochemical test outcome, any vitamin B6 supplementation should be discontinued for at least 1 week before measuring PLP concentration in blood samples.

#### Phosphoethanolamine (PEA)

Measurement of PEA are performed in urine samples. As in all urinary measurements, there are significant pre-analytical difficulties. To date, there are no data on any circadian rhythms of excretion, nor data on sample collection and preservation.

Additionally, data on reference values are very limited. A cross-sectional study demonstrated an age-related fluctuations of urinary PEA in 63 healthy individuals (31 males and 32 females) aged between 1 and 82 years old [37]. More recently, Imbard et al. reported data on 888 urine samples from patients aged between 1 and 19 years [38]. No sex-related influence was observed, but the correlation with age was confirmed. It emerged that at birth, urinary PEA values are widely distributed, and then rapidly decrease until the age of 3 years. Between the ages of 3 and 15 years, urinary PEA continues to decrease progressively, before stabilizing from the age of 15 years.

### Neurology

Altered neuronal concentration of PLP can cause vitamin B6-responsive epileptic seizures in children with HPP, which may appear immediately after birth and before signs of skeletal involvement. It is believed that the neonatal seizures are due to dysfunction of TNSALP and the resulting deficiency in the synthesis of inhibitory neurotransmitters (GABA). Additionally, studies on the mouse model of HPP show the presence of postnatal developmental defects in the hippocampus and neocortex, and the presence of seizures associated with dysregulation of purinergic signaling (reduced expression of the P2X7 purinergic receptor in the hippocampus and neocortex), and that the antiseizure effect of vitamin B6 may be due to its ability to block P2X7R [39].

Furthermore, TNSALP influences the cellular processes necessary for the synthesis of myelin and the maintenance of synaptic plasticity. Altered TNSALP activity can, therefore, affect the control of cognitive functions (memory, attention, regulation of emotions, behavior, etc.) and have effects on sleep, mood, anxiety due to the dysregulation of neurotransmitters such as GABA, dopamine, and serotonin [40].

Other neurological involvements described in literature that may be attributable to HPP include intracranial hypertension, craniosynostosis, and Arnold Chiari malformation [41].

### Dentistry

The earliest sign of dental involvement in HPP is premature (before 3–5 years of age) exfoliation of deciduous teeth, most commonly affecting the anterior teeth, starting with the mandibular primary incisors in the mild form, and with the involvement of the deciduous molars in the moderate form [42]. The exfoliation of the primary incisors occurs without inflammation [43, 44]. The primary teeth are shed with their roots intact. The first sign is often tooth mobility, which leads the patient and their family to seek advice. This sign is typically identified by a general dentist, and should promptly lead to specialist consultation.

Histological analysis suggested that the lack of cementum due to low ALP activity may be the cause of early exfoliation of primary teeth [42, 43, 45, 46]. The comparison between teeth of children with HPP and normal control–cases showed that both acellular and cellular cementum are affected, although no differences in the mineral content of dentin are observed [47]. However, cellular cementum may be deficient, while acellular cementum is almost completely absent. The collagen fibers of the periodontal ligament are not connected to the root through Sharpey's fibers: the exposed dentin and the periodontal ligament are separated by a non-fibrillar layer. There is a reduction in tooth-supporting tissues, with plaque accumulation in periodontal chambers [47].

Other common dental findings of HPP include defects in the shape, structure, and color of teeth: loss of alveolar bone, early loss of deciduous and permanent teeth without signs of periodontal inflammation, hypoplasia of enamel and dentine, thin dentinal walls, wide pulp chambers, thin and short roots, and dental caries [48].

The first teeth to be lost are the deciduous incisors, and later the molars. Deciduous molars tend towards opalescence and have large pulp chambers, wide root canals, and short roots. There is a reduced level of marginal alveolar bone. Teeth with large pulp chambers at the expense of root development that are short (taurodontism) are evident in the young permanent dentition. Defects in enamel and dentin mineralization produce alteration of tooth color from dark yellow to brown. There is a reduced level of marginal alveolar bone. Eruptive disorders with inclusion of permanent teeth are also frequent [49, 50].

Uncommon/underreported clinical signs include: delayed eruption of deciduous and permanent teeth, small bulbous crowns, cervical constrictions, and ankylosis of deciduous dentition.

Dental defects can be considered as a clinical marker of disease severity: severe phenotype is usually associated with severe disease [51]. In addition, growth impairment has been reported in children with odontohypophosphatasia [52].

### Genetics

Genetic testing (molecular analysis of the *ALPL* gene) is essential to confirm the diagnosis in case of clinical suspicion of HPP. It is also widely used prenatally to distinguish HPP from other skeletal dysplasias such as osteogenesis imperfecta [53]. Finally, the identification of pathogenic variants is crucial to establish the pattern of inheritance.

Molecular analysis of the *ALPL* gene, by direct sequencing and search for deletions, allows the detection of any pathogenic variants. Many patients with mild and non-specific clinical symptoms and reduced levels of ALP do not show specific mutations of the *ALPL* gene [10], so it is useful to sequence a specific panel of genes involved in bone fragility and muscle weakness for differential diagnosis of HPP using Next Generation Sequencing (NGS) techniques. Sequencing a specific gene panel is currently the most frequently used approach as it also allows the detection of rare deletions responsible for HPP. Although large genomic deletions of the *ALPL* gene represent no more than 3–4% of all cases identified so far, their detection is an option to follow in case of negativity to classic pathogenic variants of the coding sequence in clinically and biochemically confirmed cases [54].

The best approach to communicate to the patient the information related to the genetic nature of their pathology, inheritance and any necessary interventions for prevention and/or treatment is represented by genetic counseling. In addition, genetic counseling aims to help the patient understand the consequences of the diagnosis of a genetic disease such as HPP.

## Expert opinion

The multifaceted and not exclusive clinical manifestations of HPP often lead to erroneous diagnosis, misinterpretation of signs, and delays in the correct diagnosis [13]. This is particularly common in the moderate and mild forms, which present with signs and symptoms that are found in other, more frequent, and better known conditions. Moreover, the presenting signs of HPP may be very variable (musculoskeletal, neurological, dental), and a multidisciplinary approach is thus very helpful.

**Fig. 1:**
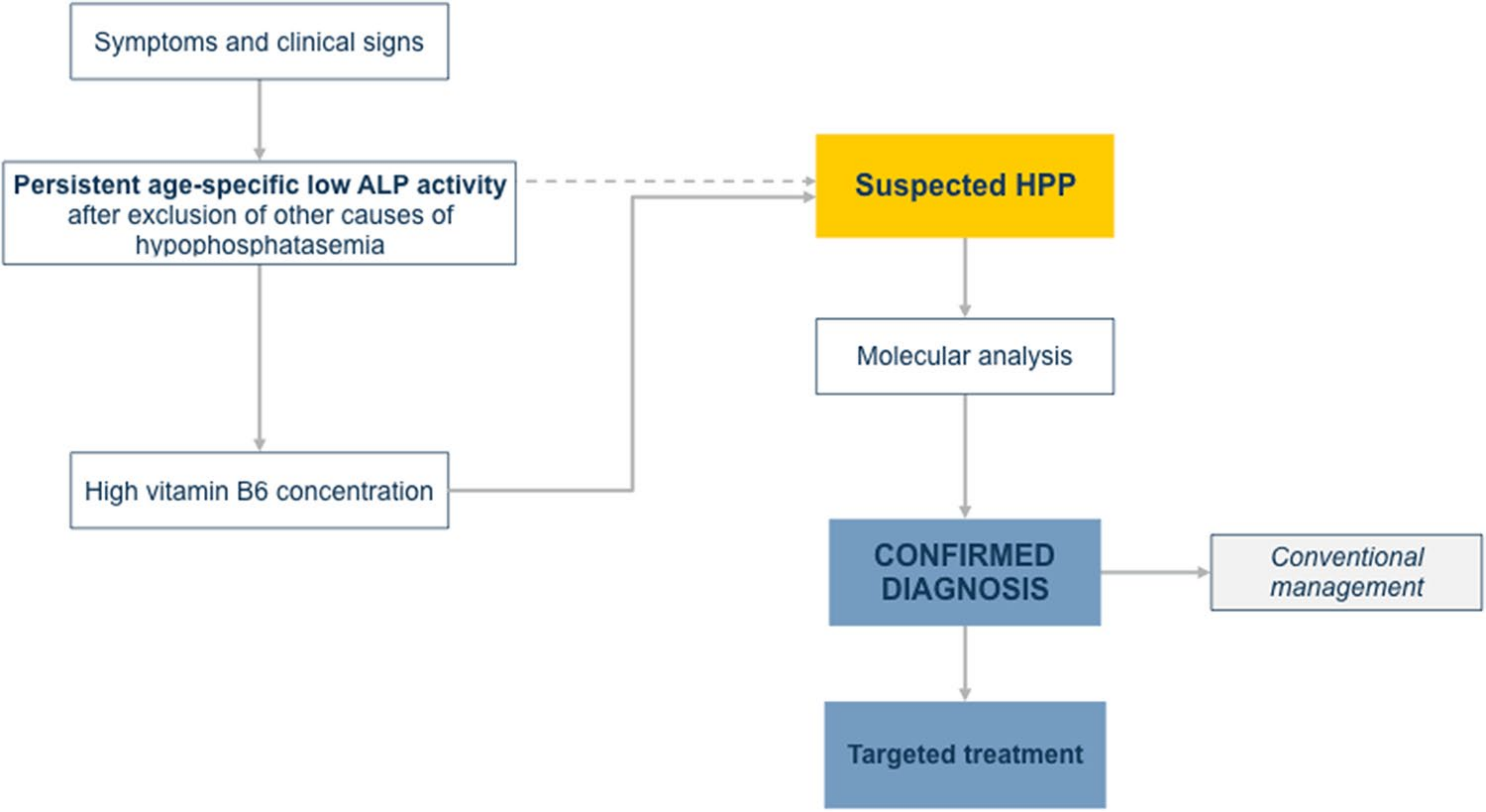
Diagnostic flowchart for children with suspected HPP. The main pathway includes the assessment of circulating ALP activity, followed by the measurement of serum vitamin B6 concentration (solid line). Whenever the latter is not feasible, the diagnosis may be confirmed performing molecular analysis directly (dotted line)

According to the previous considerations and the different clinical expertise, the components of the panel designed a diagnostic flowchart (Fig. 1). Whenever a child presents with a various combination of signs and symptoms presented in Table 1, measurement of ALP activity is mandatory. A single measurement of ALP is not sufficient to support the diagnosis of HPP. The timing of the repeated blood test is crucial, especially in severe and aggressive cases. The second blood test can be performed after 24 h or up to 10 days later, depending on the patient's clinical condition, according to medical judgment. This is particularly important in children managed on an outpatient basis without complications.

There is a strong need to accurately interpret data with ranges that take into account sex and age and that are specific for each analytical method used for the determination of ALP activity.

When a persistently low ALP activity has been documented, the presence of other conditions that may be associated with hypophosphatasemia must be considered (Table 2). After the exclusion of other forms of hypophosphatasemia, measurements of the ALP substrate are helpful to confirm the diagnostic suspect. To date, PPi measurements are not routinely performed, measurements of PLP (Vitamin B6) and PEA are available in many laboratories. It is our opinion that measurements of PLP are preferred in children and adolescents, for different reasons. Although PLP serum concentration in patients with HPP varies greatly, it is elevated in almost all cases. The availability of reference data is another important issue.

False-positive elevations are found during vitamin B6 supplementation, and are avoided if the supplementation is stopped one week before the blood collection [55].

The following step of the diagnostic pathway is represented by the molecular analysis. Before proceeding with the analysis, a genetic counseling is recommended. A condition in which counseling with a clinical geneticist is mandatory is prenatal diagnosis, when ultrasound reveals fetal skeletal abnormalities that suggest the presence of the condition. However, appropriate genetic counseling should be always provided before performing molecular genetic testing in the presence of positive clinical parameters for HPP. Genetic counseling is also very useful when receiving the results of the molecular test to define the meaning of the emerged data, and to interpret the molecular results. It is also very important to evaluate the familiarity of the pathology by extending the test to relatives of already diagnosed patients [56].

We have experiences of centers that have difficulties in providing timely results for vitamin B6, either because local laboratories do not have the measurement procedure set up, or because the time to have a result is too long. In selected cases, it is thus possible to skip this step and to perform directly the molecular analysis.

Some patients may be referred to pediatric neurology specialist in the first place, as neurological involvement may be more prominent. In this case, we selected sign and symptoms that may prompt to the suspicion of HPP (Table 3). Such signs and symptoms differ according to the levels of severity of the disease. Once the diagnosis of HPP has been ascertained, a specific neurological follow-up may be necessary in some patients. Table 4 shows the specific diagnostic workup to be done in addition for each form of the disease.

**Table 3:** Specific Red Flags according to the severity of HPP

Severe form	Moderate form	Mild form
Early seizures occurring in the first few days of life (tonic/spasms/myoclonic), typically resistant to antiseizure medications	Seizures, predominantly focal, non-responsive to antiseizure medications	Headache
Hypotonic syndrome	Headache	Sleep disturbances
Psychomotor delay	Sleep disturbances	Anxiety-depressive syndrome
Irritability	Anxiety-depressive syndrome	Cognitive disturbances (memory and attention deficit)
	Neuropathy	
	Vestibular-cochlear symptoms	
	Syncope	
	Cognitive disturbances (memory and attention deficit)

**Table 4:** Specific clinical neurological evaluation and laboratory tests for each form of the disease

Severe forms	Polysomnography EEG: findings of EEG with epileptiform/aspecific slow-wave abnormalities (even rarer normal EEG patterns) up to severe EEG patterns with burst-suppression or hypsarrhythmia Acute trial with 100 mg IV B6 during EEG recording Brain MRI
Moderate forms^a^	Standard awake EEG with video recording Neuropsychological evaluation Cognitive-psychological and QoL tests: SF36 [57] FSS test [58] DASS21 test [59] RBANS [60] VAS Pain [61] Other tests as needed (tilt test, ENT, etc.)
Mild forms^a^	Cognitive-psychological and QoL tests: SF36 [57] FSS test [58] DASS21 test [59] RBANS [60] VAS Pain [61]

*SF36* Short Form 36, *FFS* Fatigue Severity Scale, *DASS21* Depression Anxiety Stress Scales, *RBANS* Repeatable Battery for the Assessment of Neuropsychological Status, *VAS Pain* Visual Analogue Scale, *ENT* Ear Nose Throat examination

^a^At the specialist's discretion

The diagnostic process of odontohypophosphatasia involves both a clinical analysis of deciduous teeth, in which atypical tooth mobility is present in relation to age, and a radiographic analysis through orthopantomography, periapical endo-oral radiography (molars with a shell-like appearance due to wide pulp chambers and thin dentin), and cone beam computed tomography (CBCT) radiography (to evaluate the reduction of marginal bone). It is also essential to coordinate with a geneticist for genetic analysis and a pediatric endocrinologist [51].

The diagnosis of HPP in children still represents a challenge for many health providers. The diverse phenotypes that characterize the disease contribute to the diagnostic difficulties. However, the awareness of the importance of a correct interpretation of ALP activity measurements may lead to a correct differential diagnosis and to a targeted therapy. The neurological and dental involvements advocate for a multidisciplinary approach in the diagnosis of HPP.

## Data Availability

This is an expert opinion, and therefore no data have been produced.
