# Correction to: The diagnosis of hypophosphatasia in children as a multidisciplinary effort: an expert opinion

**DOI:** 10.1007/s40618-024-02403-5

**Published:** 2024-06-10

**Authors:** G. I. Baroncelli, G. Carlucci, E. Freri, M. R. Giuca, V. Guarnieri, G. Navarra, B. Toschi, S. Mora

**Affiliations:** 1 Pediatric and Adolescent Endocrinology, Division of Pediatrics, Department of Obstetrics, Gynecology and Pediatrics, University Hospital, Pisa, Italy; 2 OPT S.P.A., Soluzioni Per Il Mondo Healthcare, Milan, Italy; 3 Department of Pediatric Neuroscience, Fondazione IRCCS Istituto Neurologico Carlo Besta, Milan, Italy; 4 Unit of Pediatric Dentistry, Department of Surgical Medical Molecular Pathology and Critical Area, Dental and Oral Surgery Clinic, University of Pisa, Pisa, Italy; 5 Division of Medical Genetics, Fondazione IRCCS Casa Sollievo della Sofferenza, Foggia, Italy; 6 Section of Medical Genetics, Department of Medical and Oncological Area, University Hospital, Pisa, Italy; 7 Laboratory of Pediatric Endocrinology, Department of Pediatrics, IRCCS San Raffaele Hospital, Milan, Italy

**Correction to: Journal of Endocrinological Investigation (2024) 47:739–747** 10.1007/s40618-023-02199-w

The acknowledgement section has been incorrectly published in the original publication. The complete correct acknowledgement is given below.

This work has been made with the non-conditional contribution of Alexion. Dr. Giampiero I. Baroncelli is a representative of ERN-BOND. Dr. Elena Freri is a member of the ERN-EpiCARE network. Dr. Stefano Mora is a representative of Endo-ERN. This work is generated within the European Reference Network for Rare Bone Diseases.

The original article has been corrected.

